# Magnetic *Pycnoporus sanguineus*-Loaded Alginate Composite Beads for Removing Dye from Aqueous Solutions

**DOI:** 10.3390/molecules19068276

**Published:** 2014-06-18

**Authors:** Chih-Hui Yang, Ming-Cheng Shih, Han-Chen Chiu, Keng-Shiang Huang

**Affiliations:** 1 Department of Biological Science and Technology, I-Shou University, Kaohsiung City 82445, Taiwan; 2 The School of Chinese Medicine for Post-Baccalaureate, I-Shou University, No.8, Yida Road, Jiaosu Village Yanchao District, Kaohsiung City 82445, Taiwan

**Keywords:** *Pycnoporus sanguineus*, malachite green, dye, alginate, iron oxide nanoparticles

## Abstract

Dye pollution in wastewater is a severe environmental problem because treating water containing dyes using conventional physical, chemical, and biological treatments is difficult. A conventional process is used to adsorb dyes and filter wastewater. Magnetic filtration is an emerging technology. In this study, magnetic *Pycnoporus sanguineus*-loaded alginate composite beads were employed to remove a dye solution. A white rot fungus, *P. sanguineus*, immobilized in alginate beads were used as a biosorbent to remove the dye solution. An alginate polymer could protect *P. sanguineus* in acidic environments. Superparamagnetic nanomaterials, iron oxide nanoparticles, were combined with alginate gels to form magnetic alginate composites. The magnetic guidability of alginate composites and biocompatibility of iron oxide nanoparticles facilitated the magnetic filtration and separation processes. The fungus cells were immobilized in loaded alginate composites to study the influence of the initial dye concentration and pH on the biosorption capacity. The composite beads could be removed easily post-adsorption by using a magnetic filtration process. When the amount of composite beads was varied, the results of kinetic studies of malachite green adsorption by immobilized cells of *P. sanguineus* fitted well with the pseudo-second-order model. The results indicated that the magnetic composite beads effectively adsorbed the dye solution from wastewater and were environmentally friendly.

## 1. Introduction

Water pollution caused by the discharge of industrial wastewater that contains dyes and heavy metal effluents has become a critical environmental problem [1,2]. Most developing countries have experienced severe water shortages that are attributable to water pollution caused by rapid industrialization [3,4]. From ecological and economical perspectives, removing dyes from textile effluents is crucial. Several dye removal processes, including coagulation, flocculation, biodegradation, adsorption on activated carbon, membrane separation, ion exchange, oxidation, advance oxidation, and selective bioadsorption, have been developed. Adsorption is a simple method for removing dyes. Adsorption processes often involve passive uptake and physicochemical binding of chemical species or ions to a solid surface.

Adsorbing pollutants by using environmentally friendly materials is generally considered superior to traditional methods [5]. Numerous studies have examined magnetic polymer beads [6,7,8,9,10,11]. Magnetic separation is widely used in the fields of medicine, diagnostics, molecular biology bioinorganic chemistry, and catalysis [12,13,14]. Magnetic separation is a promising novel environmental purification technique because it produces no contaminants such as flocculants. Therefore, using magnetic beads in filtration and separation is an emerging method for treating wastewater [15,16]. Magnetic separation was proved to be an effective technique for removing dyes from manufacturing effluents [17]. Adsorption using fungus is an alternative method for treating wastewater [18,19,20,21,22,23,24]. Researchers have reported that white rot fungus (e.g., *Pycnoporus sanguineus*, *P. sanguineus*) can be used to decolorize bromophenol blue and malachite green dyes [25]. However, the immobilized fungus has limitations, such as poor mechanical strength, instability at a low pH, and cell leakage. 

**Figure 1:**
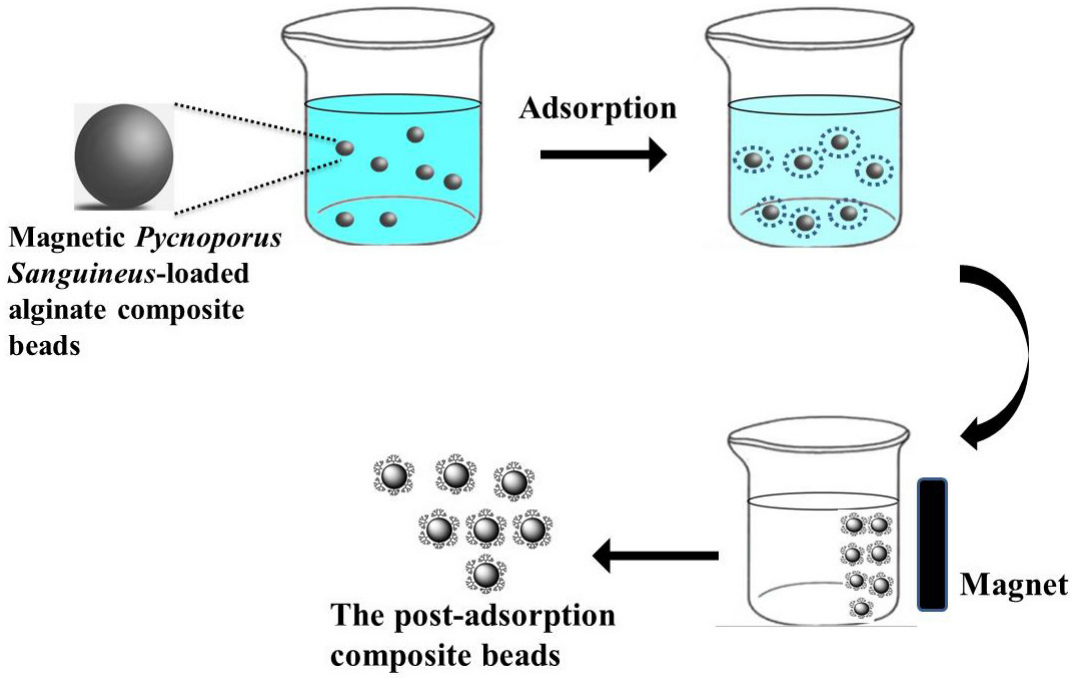
Schematic diagram of the adsorption and magnetic separation processes.

According to a review of relevant research, few studies have examined using a combination of *P. sanguineus*, magnetic beads, and pH-sensitive alginate polymers to treat wastewater. In this study, magnetic *P. sanguineus*-loaded alginate composite beads were used as adsorbents to treat an aqueous solution containing malachite green (Figure 1). In addition to adsorption capacity, batch kinetics, the effect of contact time, the effect of the initial malachite green concentration, and magnetic separation were investigated. 

## 2. Results and Discussion

### 2.1. Characterization of Magnetic P. sanguineus-Loaded Alginate Composite Beads

**Figure 2:**
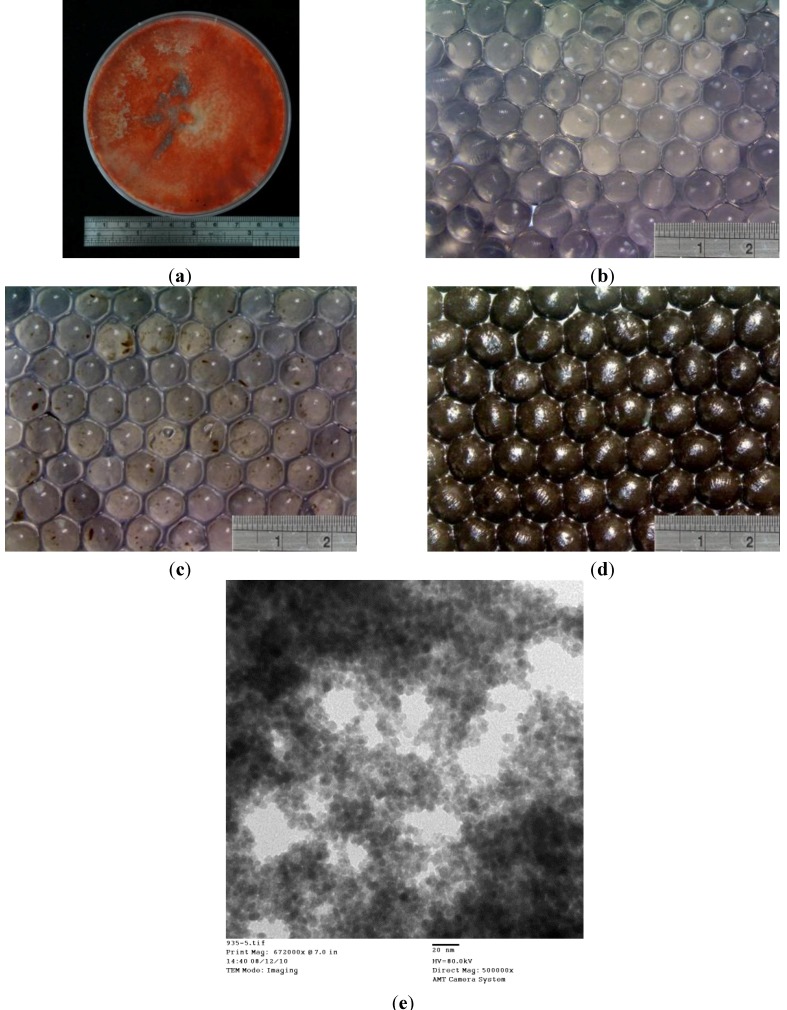
Optical microscopy images of (**a**) *P. sanguineus* in a dish, (**b**) alginate beads, (**c**) *P. sanguineus*-loaded alginate beads, (**d**) *P. sanguineus*-loaded alginate beads with magnetic iron oxide nanoparticles (e.g., magnetic *P. sanguineus*-loaded alginate composite beads), and (**e**) the SEM image of the prepared superparamagnetic nanoparticles (scale bar is 20 nm, 80 kV, 500,000×).

Figure 2 shows the morphologies of the prepared alginate beads, *P. sanguineus*-loaded alginate beads, and *P. sanguineus*-loaded alginate beads with magnetic iron oxide nanoparticles. The alginate beads were transparent particles with a mean diameter of 700 μm (Figure 2b). The *P. sanguineus*-loaded alginate beads exhibited transparent particles containing brown spots indicating the aggregation of *P. sanguineus*. The results shown in Figure 2c revealed that all alginate beads contained brown spots, suggesting that *P. sanguineus* was dispersed within the beads. The *P. sanguineus*-loaded alginate beads with magnetic iron oxide nanoparticles (Figure 2d) exhibited opaque brown and black surfaces, preventing *P. sanguineus* from being observed. The mean diameter of each bead was 800 μm. The results indicated that the relative standard deviation of composite beads shown in Figure 2d was >10%, and the beads were elliptical in shape. We attribute the elliptical shape to the lower concentration of the Na-alginate solution (0.9 wt %) used in the *P. sanguineus*-loaded alginate beads with magnetic iron oxide nanoparticles relative to that used in the alginate beads (2 wt %). The beads were hydrogels. When the concentration of the Na-alginate solution was below 1 wt %, the polymer beads exhibited a loose structure and became nonspherical. In addition, the prepared magnetic nanoparticles were shown in Figure 2e. The magnetic properties and aggregation status of magnetite nanoparticles have been described in the literature [26]. It provide clues for optimal the prepared magnetite nanoparticles. 

### 2.2. Removal of Dye from an Aqueous Solution

**Figure 3:**
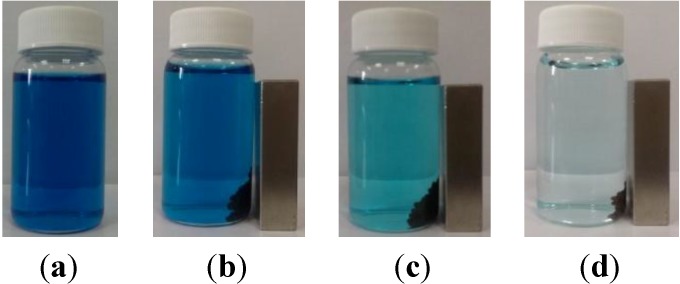
Photographs of malachite green dye treatment using magnetic *P. sanguineus*-loaded alginate composite beads: (**a**) original solution (**b**) baseline, (**c**) after 100 min of adsorption, and (**d**) after 1440 min adsorption.

The prepared magnetic *P. sanguineus*-loaded alginate composite beads (0.05 mg) were added to 16.65 mL of a 10 mg/L malachite green solution at room temperature (approximately 298 K) to evaluate the efficiency of dye adsorption. To observe the color change of the solution in a vial, a magnet was used to concentrate the composite beads. Figure 3a,b show the initial picture. When the composite beads were aggregated using a magnet, the solution was deep blue in color. The purified solution and postadsorption composite beads could be separated easily when the iron oxide nanoparticles were used. The magnet was removed to enable adsorption to proceed for 100 min. The composite beads were aggregated using the magnet again, revealing that the composite beads caused the deep blue color of the solution to fade, and a light blue color was observed (Figure 3b). The magnet was then removed to enable 1,440 min of adsorption to proceed. Subsequently, the composite beads were aggregated using the magnet, and the solution became nearly colorless (Figure 3c). The results revealed that the prepared magnetic *P. sanguineus*-loaded alginate composite beads adsorbed malachite green gradually over time. We speculate that the mechanism of biosorption is based on the biosorption of dyes by *P. sanguineus* [27]. 

### 2.3. Effect of Initial Dye Concentration and Contact Time

**Figure 4:**
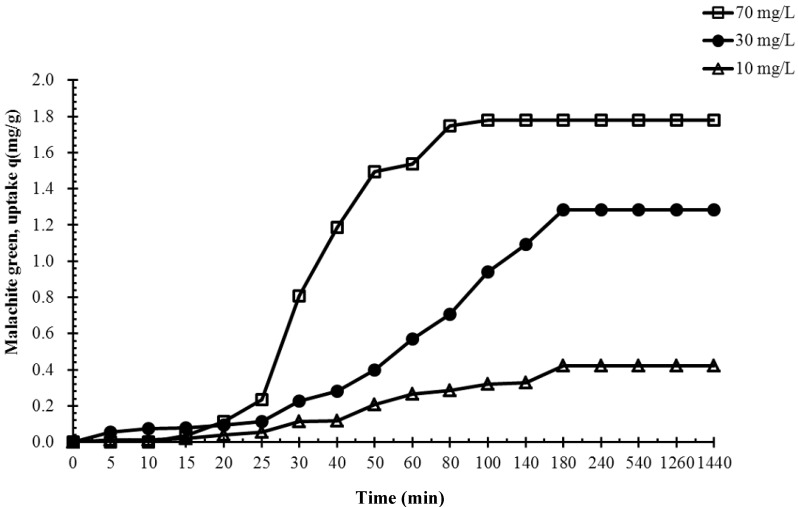
Effects of initial dye concentration on the biosorption of the malachite green dye by immobilized cells of *P. sanguineus* (temperature: approximately 25 °C).

To evaluate the adsorption effect, three concentrations of malachite green solutions were tested at room temperature (approximately 298 K), as shown in Figure 4. 

The results indicated that the maximal uptake capacities were 0.4, 1.35 and 1.8 mg/g for the 10, 30, and 70 mg/L malachite green solutions, respectively. The time required to reach equilibrium differed according to the concentration of the malachite green solution; specifically, equilibrium was reached after the 10 mg/L malachite green solution was adsorbed for 180 min, after the 30-mg/L malachite green solution was adsorbed for 180 min, and after the 70 mg/L malachite green solution was adsorbed for 90 min. The results revealed that each adsorption rate increased before the maximal uptake capacity was reached. We infer that the prepared composite beads feature high adsorption and a high adsorption rate at high concentrations. 

### 2.4. Effect of pH on Adsorption

**Figure 5:**
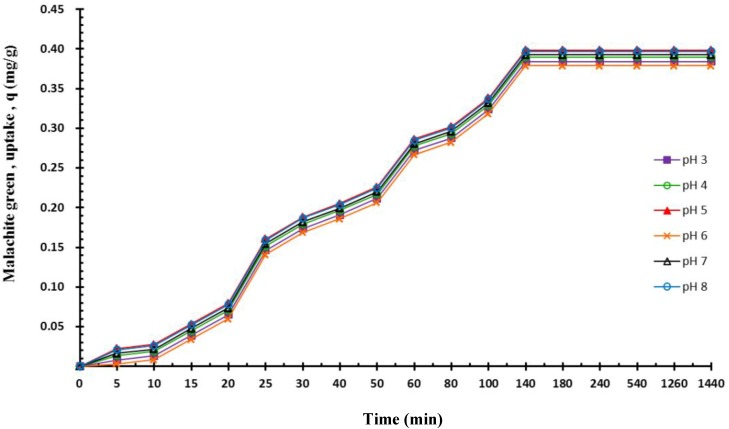
Effects of pH values on the biosorption of the malachite green dye (50-mL centrifugal tube, agitation speed = 125 ppm).

The effect of pH values ranging from 3 to 8 on dye adsorption (Figure 5) was investigated using 16.65 mL of a 10 mg/L malachite green solution, 0.05 mg of composite beads, and a reaction temperature of 298 K. The results indicated that pH variations did not influence the dye adsorption capacity. No significant differences were observed among pH values. The results indicated that, in an acidic environment, the alginate polymer beads adequately protected *P. sanguineus*. The results evidenced that the magnetic *P. sanguineus*-loaded alginate composite beads effectively adsorbed dye pollutants.

### 2.5. Kinetics Studies

**Figure 6:**
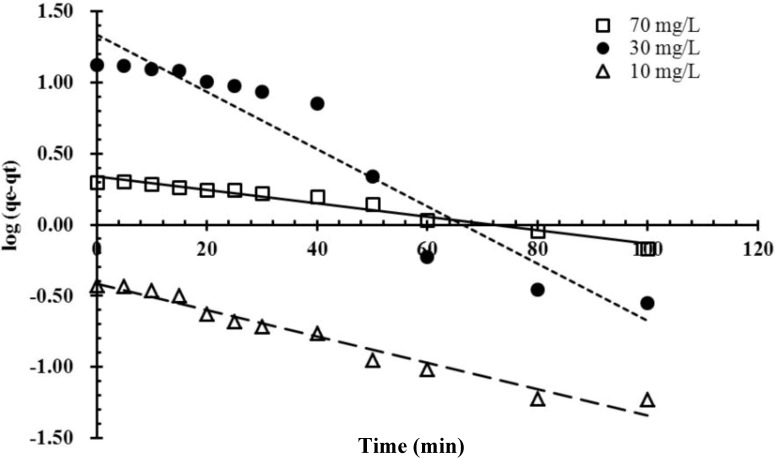
Linearized pseudo-first-order kinetics plot of the biosorption of the malachite green dye onto immobilized cells of *P. sanguineus* at various concentrations of composite beads.

Kinetics experiments of malachite green biosorption by immobilized cells of *P. sanguineus* were conducted, and the experimental data were fitted to the pseudo-first-order and pseudo-second-order kinetic models. The best-fit model was selected based on the values of the linear regression correlation coefficient *R*^2^. The adsorption of malachite green was plotted according to Equation (4) and is shown in Figure 6. The pseudo-first-order constants and correlation coefficient *R^2^* were calculated based on Figure 6 and are listed in Table 1. The high *R*^2^ values confirm that the pseudo-first-order kinetics model suitably represented all adsorption data. Furthermore, the calculated values of adsorption capacity *qe* obtained from the pseudo-first-order model were reasonably close to the experimental *qe* values in the case of pseudo-first-order kinetics. The rate of a pseudo-second-order reaction depends on the amount of solute adsorbed on the surface of the adsorbent and the amount adsorbed at equilibrium [28]. When pseudo-second-order kinetics is applicable, the plot of *t*/*qt* against *t* for Equation (5) shows a linear relationship from which *qe* and *k*_2_ can be determined based on the slope and the intercept, respectively. As shown in Table 2, the results indicated that the experimental data were not consistent with the pseudo-second-order kinetic model. 

**Table 1:** Kinetic pseudo-first-order model parameters of the biosorption at various concentrations of composite beads.

Co mg/L	T (K)	Pseudo-First-Order		
		K_1_	*q_e_*	R^2^
70	298	0.080	1.40	0.981
30	298	0.045	0.26	0.912
10	298	0.024	0.68	0.991

**Table 2:** Kinetic pseudo-second-order model parameters of the biosorption at various concentrations of composite beads.

Co mg/L	T (K)	Pseudo-Second-Order		
		K_2_	*q_e_*	R^2^
70	298	0.004	2.214	0.984
30	298	0.242	0.275	0.760
10	298	0.023	0.420	0.928

**Figure 7:**
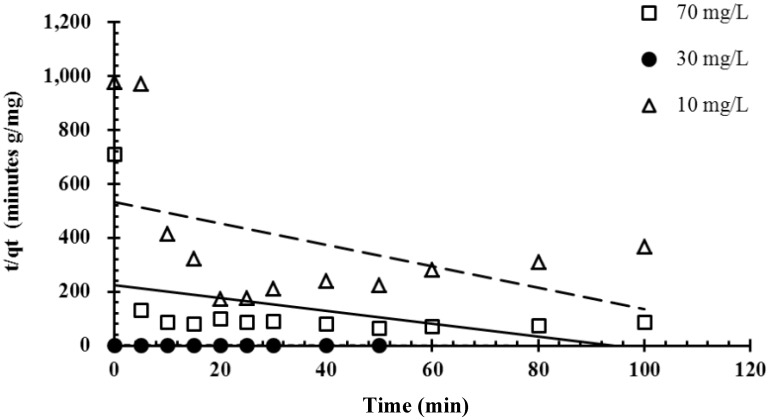
Linearized pseudo-second-order kinetics plot of the biosorption of malachite green onto immobilized cells of *P. sanguineus* at various concentrations of composite beads.

Figure 6 and Figure 7 show a comparison of pseudo-first-order and pseudo-second-order kinetic models of malachite green adsorption onto magnetic *P. sanguineus*-loaded alginate composite beads at various initial dye concentrations. All experimental data on malachite green adsorption onto magnetic *P. sanguineus*-loaded alginate composite beads were consistent with the pseudo-first-order model and exhibited high correlation coefficients. Theoretically, the Lagergren pseudo-first-order equation indicates that the logarithm of the difference between the equilibrium and the monitored adsorbed amount should be a linear function of time. This equation is closely associated with the model of one-site occupancy adsorption kinetics governed by the rate of the surface reaction. The results shown in Figure 6 indicated that the adsorption process observed in this study was consistent with this model. The Lagergren pseudo-first-order equation was recently generalized to two-site-occupancy adsorption and called the pseudo-second-order kinetic equation. The main assumption of the pseudo-second-order kinetic model is that the sorption capacity is proportional to the number of active sites occupied on the sorbent. According to relevant literature, pseudo-second-order kinetics is more applicable to dye adsorption than other kinetic models because the effects of transport phenomena and chemical reactions are often experimentally inseparable. Figure 7 shows that the adsorption process was not two-site-occupancy adsorption. 

## 3. Experimental

### 3.1. Microorganism and Production Medium

*P*. *sanguineus* was obtained from the Bioresource Collection and Research Center, Food Industry Research and Development Institute, Taiwan. The fungus was maintained through weekly transfer on malt extract agar slants incubated at 308 K for 7 d and was subsequently stored at 277 K until required. A 500-mL production medium consisting of 0.15 g/mL of glucose, 0.3 g/mL of peptone, and 24 g/mL of malt extract was autoclaved at 394 K (150 kN/m^2^) for 20 min. 

### 3.2. Preparation of Iron Oxide Nanoparticles

Ferrous chloride 4-hydrate (FeCl_2_•4H_2_O, 98%), iron (III) chloride hexahydrate (FeCl_3_•6H_2_O, 99%), and sodium hydroxide (NaOH) were purchased from Sigma (Uni-onward, Taiwan), J. T. Baker (Uni-onward, Taiwan), and Alfa Aesar (Uni-onward, Taiwan), respectively. A 2 N FeCl_2_•4H_2_O ferro solution (1 mL) and a 2 N FeCl_3_•6H_2_O ferric solution (4 mL) were mixed. After 15 min of constant stirring, a ferro-ferric solution was obtained. Subsequently, 5 mL of glycine was added to the ferro-ferric solution, and the solution was stirred for 15 min. A 20% NaOH solution (9 mL) was added dropwise to the ferro-ferric-glycine solution, and the solution was stirred at room temperature (approximately 298 K). After 5 min, black iron oxide nanoparticles were observed based on the coprecipitation of both ferrous cations and ferric cations. The particles were then collected by conducting centrifugation and washed several times with 30 mL of distilled deionized water to remove any alkali. Similar synthetic methods have been described [29,30,31]. 

### 3.3. Preparation of Calcium-Alginate Beads

Ca-alginate beads were prepared by dropping 3 mL of a 2 wt % Na-alginate solution (Na-alginate powder from a brown algae source, 300–400 cps, purchased from Sigma (Uni-onward, Taiwan)) into 50 mL of a 20% CaCl_2_ solution at room temperature (approximately 298 K). The beads were stirred slowly for 30 min, collected through filtration, and washed with sterile deionized water. The washed beads were collected and stored at room temperature (approximately 298 K). Similar synthetic methods have been described [32,33,34,35].

### 3.4. Preparation of Magnetic P. sanguineus-Loaded Alginate Composite Beads

A cell suspension was prepared by inoculating a stock culture of *P. sanguineus* onto malt extract agar plates. The mycelium mat that formed was scraped from the plate by using a sterile blade and mixed with 50 mL of a sterile Tween 20 (purchased from Sigma (Uni-onward, Taiwan)) solution before placing it in a sterile sampling bottle (100 mL). The sampling bottle was vortexed for 5 min to distribute the mycelium evenly in the liquid. Subsequently, 15 mL of the cell suspension was inoculated into an Erlenmeyer flask containing 135 mL of the production medium. The flask was incubated on a rotary shaker operating at 150 rpm at 303 K for 66 h. The harvested culture broth was centrifuged at 4,500 rpm and 298 K for 4 min. 

The magnetic *P. sanguineus*-loaded alginate composite beads were prepared by dropping a mixture comprising 2 mL of the 2 wt % Na-alginate solution, 1 mL of iron oxide nanoparticles, and 2 mL of a 2% *P. sanguineus* solution into a 50-mL 20% CaCl_2_ solution at room temperature (approximately 298 K). The beads were stirred slowly for 30 min, collected through filtration, and washed with sterile deionized water. The washed beads were collected and stored at room temperature (approximately 298 K).

### 3.5. Preparation of Dye Solutions

The stock solution of a malachite green dye (chemical formula: C_23_H_25_N_2_•0.5C_2_H_2_O_4_; λ*_max_* = 620; purchased from Sigma (Uni-onward, Taiwan)) were prepared by dissolving 1 g of the dye in 1 L of sterile deionized water. The stock solution was then successively diluted to the desired initial concentrations, namely 10 mg/L, 30 mg/L, and 70 mg/L. 

### 3.6. Kinetic Studies

Predicting batch kinetics can facilitate the design of adsorption systems because kinetics describes the uptake rate of adsorbents, thereby enabling the adsorption mechanism to be predicted [36,37,38,39,40]. 

#### 3.6.1. Pseudo-First-Order Kinetic Model

The pseudo-first-order kinetic model has been used extensively to predict sorption kinetics. The model proposed by Langergren [41] is expressed as follows:

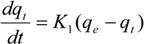
(1)


After definite integration is established by applying the boundary conditions *t* = 0 to *t* = *t* and *q_t_* = 0 to *q_t_* = *q_t_*, the integrated form of Equation (1) can be expressed as follows [40]:

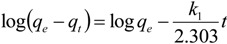
(2)
where *q_e_* and *q_t_* are the amounts of adsorbate adsorbed (mg/g) at equilibrium and time *t* (min), respectively, and *k*_1_ is the adsorption rate constant of the pseudo-first-order equation (min^−1^). The values of log(*q_e_ −* *q_t_*) were linearly correlated with *t*. The plot of log(*q_e_* − *q_t_*) *versus* *t* shows a linear relationship from which *K*_1_ and *q_e_* can be determined based on the slope and intercept of the plot, respectively.

#### 3.6.2. Pseudo-Second-Order Kinetic Model

The pseudo-second-order equation is expressed as follows [28]:

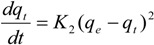
(3)


Integrating Equation (4) for the boundary conditions *t* = 0 to *t* = t and *q_t_* = 0 to *q*_t_ = *q_t_* yields

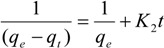
(4)
which is the integrated rate law for a pseudo-second-order reaction. Equation (4) can be rearranged to obtain Equation (5), which has a linear form:

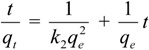
(5)
where *k*_2_ is the rate constant of the pseudo-second-order equation (g/mg min). The linear plot of *t*/*q_t_ versus* *t* indicates that 1/*q_e_* is the slope and 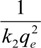 is the intercept.

## 4. Conclusions

In this study, alginate composite beads containing magnetic iron oxide and activated *P. sanguineus* were prepared to selectively remove a dye from synthetic wastewater. The results indicated that the immobilized cells of *P. sanguineus* can be used as an adsorbent to remove malachite green. In addition, no significant difference was observed between the adsorption capacity of malachite green and pH variations. This high pH range tolerance advance the application of the prepared composite beads in acidic conditions. The magnetic properties of the composite beads enabled them to be separated from the effluent by using a simple magnetic field, thus facilitating the development of a simple process for remediating water polluted with dyes.
